# Editorial: Maternal nutrition, gut microbiota, and endocrine programming in early life

**DOI:** 10.3389/fendo.2026.1859900

**Published:** 2026-05-12

**Authors:** Gratiela Gradisteanu Pircalabioru, Mara Ioana Ionescu, Robin Bernstein

**Affiliations:** 1 Department of Botany and Microbiology, Faculty of Biology, University of Bucharest, Bucharest, Romania; 2 eBio-Hub Centre of Excellence in Bioengineering, National University of Science and Technology Politehnica Bucharest, Bucharest, Romania; 3 Department of Functional Sciences, Carol Davila University of Medicine and Pharmacy, Boulder, CO, United States; 4 Department of Pediatrics, Marie Curie Emergency Children's Hospital, Bucharest, Romania; 5 Health and Society Program, Institute of Behavioral Science, University of Colorado, Boulder, CO, United States

**Keywords:** early life, endocrine programming, gut microbiota, maternal nutrition, microbiome

Maternal nutrition during pregnancy is a major determinant of offspring health, extending beyond fetal growth and birth outcomes. The intrauterine environment is now recognized as a critical window in which nutritional, metabolic, microbial, and immune signals shape endocrine development and lifelong disease risk. A key mediator of this process is the maternal gut microbiota, a dynamic ecosystem influenced by diet, metabolic status, medication use, and inflammation. By affecting nutrient metabolism, immune maturation, endocrine signaling, and the production of bioactive metabolites, the maternal microbiota may provide an important mechanistic link between maternal lifestyle and offspring health.

**Figure 1:**
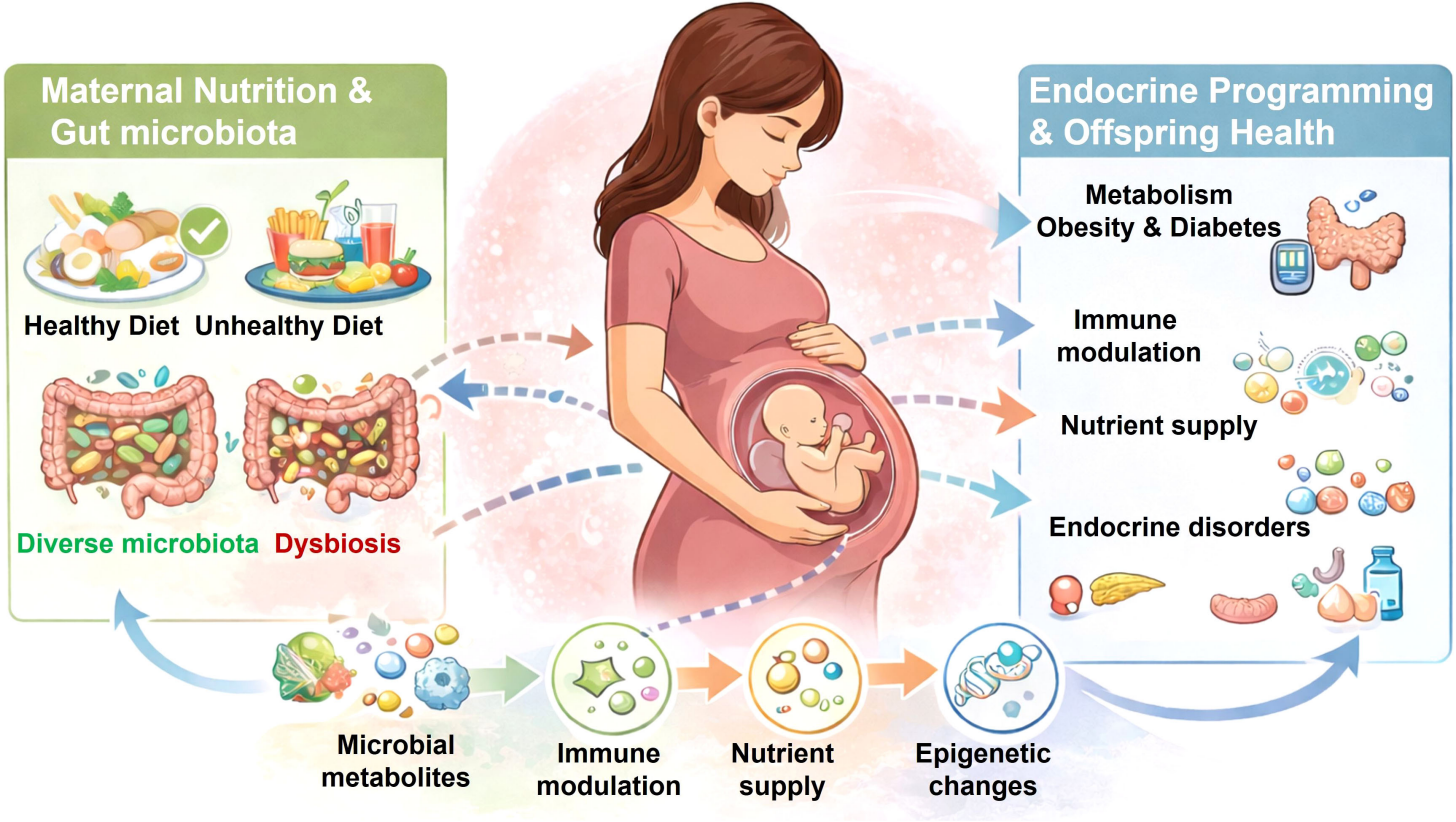
Maternal nutrition, gut microbiota, and endocrine programming in early life.

These interconnected pathways are summarized in **Figure 1**, which illustrates how maternal diet-driven alterations in gut microbiota may influence offspring endocrine development through microbial metabolites, immune modulation, nutrient availability, and epigenetic mechanisms.

This Research Topic explores how maternal nutrition shapes maternal and neonatal microbiota and how these changes influence offspring endocrine development. While links between maternal diet, obesity, gestational diabetes, antibiotic exposure, and later cardiometabolic risk in children are increasingly recognized, the underlying mechanisms remain incompletely defined. By integrating perspectives from endocrinology, microbiology, nutrition, developmental biology, and translational medicine, this Research Topic offers new insight into how maternal-offspring interactions may drive the intergenerational disease transmission.

A central theme across the Research Topic is the influence of maternal nutritional and metabolic status on fetal development. In the study by Stunes et al., “*Maternal fat-soluble vitamin trajectories and infant birth weight in individuals with overweight or obesity*”, the authors examined maternal concentrations of vitamins A, D, and E during pregnancy in individuals with overweight or obesity. They observed significant declines in retinol and 25(OH)D, alongside an increase in α-tocopherol over the course of pregnancy. Vitamin A insufficiency, vitamin D deficiency or insufficiency, and macrosomia were common in this high-risk cohort. Their findings suggest that micronutrient dynamics during gestation may not be adequately captured by single time-point measurements. More broadly, these findings reinforce the view that maternal nutrition should be considered not only in terms of energy intake, but also in relation to micronutrient sufficiency, metabolic context, and the downstream biological systems that may shape offspring endocrine health.

Maternal metabolic disorders further contribute to the developmental programming. In The impact of maternal gestational diabetes mellitus on cardiac structural and functional parameters in infants, Deng et al. show that gestational diabetes is associated with subtle but significant sex-specific alterations in infant cardiac structure. These findings suggest that maternal metabolic dysregulation can imprint early-life measurable physiological changes even in the absence of overt pathology. This study extends the concept of endocrine programming and emphasizes the importance of considering cardiovascular development as part of the endocrine-metabolic phenotype. It also highlights the possibility of sex-specific programming effects, which deserve further mechanistic study.

The Research Topic also expands the developmental window during which maternal exposures may influence offspring disease susceptibility. In their systematic review and meta-analysis, De Pasquale and Harrison examined early-life antibiotic exposure and type 1 diabetes risk across preconception, prenatal, neonatal, and postnatal periods. Notably, they found that maternal preconception exposure to specific antibiotic classes was associated with increased odds of type 1 diabetes in the offspring, whereas prenatal and postnatal exposures were not significantly associated with risk. These findings are especially intriguing because they point to a potentially modifiable maternal factor active during preconception. The well-established effects of antibiotics on gut microbial composition and metabolite production, this work raises important questions about whether maternal microbiome disruption prior to conception may alter immune and endocrine programming in the offspring. It also underscores the need for future studies to account for timing, antibiotic class, microbial recovery, and host susceptibility when investigating the developmental origins of endocrine disease.

Epigenetic mechanisms are increasingly recognized as key mediators of maternal effects on offspring development, as in the study of González-González et al.- “*Infants exposed to maternal type 1 diabetes: intrauterine epigenetic modifications and neurological development*”. The authors show that exposure to maternal type 1 diabetes is associated with differential DNA methylation in cord blood at neurodevelopment-related genes, alongside impaired cognitive, language, and motor outcomes at two years. While focused primarily on neurodevelopment, this study demonstrates that maternal metabolic disease can induce detectable intrauterine epigenetic marks with lasting postnatal consequences. These findings align closely with current models of endocrine and metabolic programming, in which altered maternal metabolism and inflammation reshape fetal gene regulation, developmental pathways, and long-term disease risk, illustrating the value of integrating rather than viewing endocrine, neurological, and epigenetic perspectives separately.

Beyond bacteria, the maternal-offspring microbial axis may also involve fungal communities and immune-dependent microbial interactions. In “*Altered gut fungal microbiota and associated mycotoxins in juvenile rat offspring induced by maternal immune activation with Poly I:C*”, Zhong et al. report that maternal immune activation induces sex-specific changes in offspring gut fungal microbiota and mycotoxin-associated metabolites, accompanied by behavioral deficits. This study expands the conceptual framework of the maternal microbiome beyond bacterial dysbiosis alone using an animal model centered on neurodevelopmental outcomes. Results suggest that immune activation during pregnancy may reshape offspring microbial ecosystems in ways that influence metabolism, signaling pathways, and tissue development. These findings reinforce the interconnected nature of endocrine, immune, and microbial networks in early life.

Collectively, these studies demonstrate that maternal nutrition and metabolic status shape offspring health through integrated microbial, immune, metabolic, and epigenetic pathways. These effects are often subtle, sex-specific, and detectable before disease becomes clinically evident, emphasizing the need for sensitive biomarkers and long-term follow-up. They also position the maternal microbiota as an active mediator of signals transmitted to the fetus and newborn. Important gaps still remain, including the need for mechanistic studies, predictive biomarkers, and targeted interventions such as diet-based or microbiome-informed strategies to reduce intergenerational endocrine disease risk.

This graphical abstract illustrates the interconnected pathways linking maternal diet, gut microbiota composition, and offspring health outcomes. Maternal nutritional status influences gut microbiota diversity, with healthy diets promoting a diverse microbiota and unhealthy diets contributing to dysbiosis. These microbial states affect the production of bioactive metabolites, which in turn modulate immune function, nutrient availability, and endocrine signaling during fetal development. Through these integrated pathways—including microbial metabolites, immune modulation, nutrient transfer, and epigenetic regulation—the maternal environment shapes fetal programming. These early-life influences contribute to offspring metabolic health, immune function, and risk of endocrine-related disorders later in life. Artificial intelligence (AI) tools were used to assist in the creation of this graphical abstract. The authors take full responsibility for the content and interpretation

